# Analysis of roasted peanuts based on GC–MS combined with GC–IMS

**DOI:** 10.1002/fsn3.3882

**Published:** 2023-12-07

**Authors:** Liangchen Zhang, Puxiang Shi, Jian Sun, Mengxi Xie, Haixin Wang, Taiyuan Shi, Miao Yu

**Affiliations:** ^1^ Institute of Food and Processing, Liaoning Academy of Agricultural Sciences Shenyang China; ^2^ Institute of Sandy Land Management and Utilization of Liaoning Fuxin China; ^3^ Department of Food Science Shenyang Agricultural University Shenyang China

**Keywords:** GC–IMS, GC–MS, roasted peanut, volatile flavor substances

## Abstract

The present study used gas chromatography–mass spectrometry (GC–MS) and gas chromatography–ion mobility spectrometry (GC–IMS) to separate and identify the characteristic volatile flavor substances in 30 roasted peanut samples. GC–MS identified 59 volatile compounds, and GC–IMS detected 61 volatile flavor substances. The 30 peanut varieties were then divided into four groups on the basis of their volatile flavor substances using principal component analysis (PCA), and a fingerprint profile of the varieties' volatile characteristics was established from information peaks identified in the spectra. Descriptive sensory analysis (DSA) was performed to distinguish differences in flavor attributes between roasted peanut varieties. Partial least squares regression (PLSR) was performed with the volatile flavor content of roasted peanuts as the independent variable and the flavor attribute score as the dependent variable. These findings provide a basis for predicting the appeal of roasted peanuts based on their composition and demonstrate a potential avenue for improving food flavor quality.

## Graphical abstract

The present study used gas chromatography–mass spectrometry (GC–MS) and gas chromatography–ion mobility spectrometry (GC–IMS) to separate and identify the characteristic volatile flavor substances in 30 roasted peanut samples. These findings provide a basis for predicting the appeal of roasted peanuts based on their composition and demonstrate a potential avenue for improving food flavor quality.

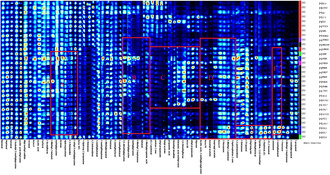

## 1 INTRODUCTION

Peanut (*Arachis hypogaea* L.) is a high‐yielding cash crop used for both food and oil (Isanga & Zhang, 2007). The composition of food flavor substances is complex, and a given food includes a variety of flavor components (Spence, 2021). Although flavor components make up a small percentage of the overall product, they contribute greatly to the taste of food. These compounds exhibit a degree of thermal instability/volatility and are sensitive to acids and bases. Roasting, steaming, and microwave‐assisted processing methods can alter the fats and proteins in food, producing volatile components. Studies that examined flavor after roasting or other processing methods found that the flavor substances of treated peanuts were composed mainly of volatile compounds (Toomer, 2018). When peanut kernels undergo high‐temperature roasting, volatile flavor components can be produced by a variety of reactions, including the Maillard reaction, lipid oxidation, and protein degradation, with the Maillard reaction being the main method (Dun et al., 2019; Liu et al., 2011).

Currently, the methods used to detect flavor substances with electronic nose coupling, full two‐dimensional gas chromatography–mass spectrometry (GC × GC/TOFMS), gas chromatography olfactometry (GC–O), high‐performance liquid chromatography (HPLC), headspace solid‐phase microextraction, and gas chromatography–mass spectrometry (GC–MS) (Jia et al., 2021). The above technologies can achieve qualitative and quantitative analysis of multicomponent volatile flavor compounds. Food flavor is a complex multicomponent system, and simply measuring one or several volatile flavor compounds makes it difficult to comprehensively reflect the internal interaction relationships of different volatile flavor substances in food. Moreover, these technologies are limited by sample pretreatment procedures and sample type requirements, and cannot meet the requirements of rapid detection and analysis (Teng et al., 2019). GC–IMS is a new technique that couples gas chromatography and ion mobility spectrometry to perform fingerprinting of volatile organic compounds in unprepared liquid or solid samples (Zhao et al., 2021). This technology has the characteristics of fast response, high sensitivity, and good stability. So far, it has been applied in food classification and adulteration, odor detection, monitoring of processing, and evaluation of aroma changes during storage (Li et al., 2021). However, this technique has not been fully explored for analysis of roasted peanut volatile flavor components; in particular, GC–IMS fingerprinting of volatile components from different varieties of roasted peanuts has not previously been reported in the published literature.

Liaoning Province in China is an important production area for high‐quality peanuts in China. Peanuts from the region have a low aflatoxin infection rate and high protein and soluble sugar contents, making them particularly suitable for food production. Here, we aimed to identify and catalog the volatile compounds that underlie the flavor of roasted peanuts, using 30 peanut varieties from Liaoning Province. Peanuts were roasted, and GC–MS was used to identify and quantify the volatile flavor components contained within each array. The study provides useful data for identifying peanut varieties and origins, while also contributing to our understanding of volatile flavor components and to quality control of processed peanut products.

## 2 MATERIALS AND METHODS

### 2.1 Peanut materials and reagents

Peanuts used in this study were provided by the Peanut Research Institute of Liaoning Province. Samples (moisture content < 10%) were harvested and immediately stored in airtight containers at 4°C until processing. The variety names and harvest times are displayed in Table 1.

**TABLE 1:** Thirty varieties of roasted peanuts.

No.	Peanut variety	Code	Variety	Harvest date	Initial moisture content/%	Moisture content after roasting/%
1	LuHua965	LH965	Virginia	10/10/2021	9.67	3.94
2	BaiSha	BS	Runner	10/05/2021	9.24	3.01
3	LiaoHua618	LH618	Runner	10/10/2021	9.45	3.24
4	ShenHua5	SH5	Valencia	09/30/2021	8.94	3.07
5	JitianHua1	JTH1	Spanish	09/30/2021	9.66	4.01
6	YuHua19	YH19	Runner	10/10/2021	9.12	3.91
7	Ji572	J572	Runner	10/11/2021	9.17	3.15
8	XinHua5	XH5	Runner	10/11/2021	9.34	3.09
9	WeiHua29	WH29	Virginia	10/05/2021	9.87	3.97
10	WeiHua23	WH23	Virginia	10/07/2021	8.47	3.07
11	JiHua19	JH19	Valencia	09/29/2021	9.66	3.67
12	FuHua29	FH29	Runner	10/11/2021	9.41	3.60
13	JiNongHua12	JNH12	Runner	10/11/2021	9.47	3.51
14	ShenHua1	SH1	Runner	09/27/2021	9.47	3.27
15	JiHua1336	JH1336	Runner	10/12/2021	9.12	3.07
16	PuHua66	PH66	Spanish	10/10/2021	8.25	3.01
17	YuHua37	YH37	Runner	10/09/2021	8.77	3.78
18	FuHua30	FH30	Runner	10/09/2021	9.01	3.97
19	YuHua18	YH18	Runner	09/30/2021	8.74	3.05
20	JiNongHua6	JNH6	Spanish	10/11/2021	9.21	3.91
21	YuHua65	YH65	Valencia	10/10/2021	9.47	3.67
22	FuHua23	FH23	Runner	10/08/2021	9.26	3.77
23	JiHua18	JH18	Runner	10/08/2021	9.64	3.83
24	HuaYu962	HY962	Runner	10/11/2021	8.41	3.09
25	FuHua27	FH27	Runner	10/11/2021	9.66	3.83
26	KaiNong308	KN308	Virginia	10/09/2021	9.73	3.86
27	FuHua17	FH17	Runner	09/30/2021	8.90	3.61
28	XinHua17	XH17	Runner	10/10/2021	9.02	3.77
29	FuHua22	FH22	Runner	10/10/2021	9.14	3.64
30	LiaoHua917	LH917	Runner	10/11/2021	9.87	3.74

All chemicals and solvents used for analysis were analytical grade and purchased from Sigma–Aldrich or Solarbio (Beijing Solarbio Science & Technology Co., Ltd).

### 2.2 Methods

#### 2.2.1 Pretreatment of roasted peanut

Each variety of peanut was shelled. Approximately 1000 g of kernels (no mold or damage) were weighed and then spread evenly in a single layer on a baking tray; the oven (HLK–204E, Hongling) temperature was set to 160°C and baked for 30 min, then the baking tray every was shaken 10 min to evenly heat the peanuts. Peanuts were then removed from the oven and cooled at room temperature to 20°C.

#### 2.2.2 GC–MS analysis

Samples were analyzed by GC–MS using a 7890A‐5975C gas chromatograph–mass spectrometer (Agilent Technologies).

One hundred grams of roasted peanuts were crushed, and 5 g of the paste was placed in a 20‐mL headspace vial. Ten microliters of 2‐methyl‐3‐heptanone internal standard solution was added to the vial. The mixture was then preheated at 60°C for 20 min. An aged extraction head (10 mm/80 μm DVB/CAR/PDMS; Agilent Technologies) was inserted into the vial for 30 min. The sample vial was then sorbed for 30 min.

The GC–MS settings were as follows: sample inlet temperature, 250°C; carrier gas, helium (purity ≥99.999%); constant flow mode; flow rate, 1.5 mL/min, and no split injection. The temperature ramp‐up procedure was 40°C for 3 min, 3°C/min to 150°C, and 8°C/min to 230°C for 5 min.

The mass spectrometry parameters were as follows: interface temperature, 250°C; ion source temperature, 230°C; quadrupole temperature, 150°C; electron ionization source, electron energy 70 eV; mass scan range, *m*/*z* 30–500; and solvent delay, 3 min.

Each sample was injected by rapidly inserting the completed SPME (solid‐phase microextraction) injection needle (50/30 μm DVB/CAR/PDMS; Supelco) into the GC inlet and pushing out the extraction head to desorb for 15 min while simultaneously starting the GC for data acquisition.

Components separated by GC–MS were identified by searching the NIST 14.0 database (National Institute of Standards and Technology), selecting a substance with a positive and negative matching degree greater than 80 for confirmation, using 2‐methyl‐3‐heptanone (0.408 mg/mL) as the internal standard, and using the internal standard method for quantification. Contents of target compounds were calculated using the following formula:

$$m_{i}=\frac{A_{i}}{\left(\frac{A_{s}}{m_{s}}\right)\times m}$$

*m*_i_: target compound content (μg/10 mL),*A*_i_: peak area of the target compound,*A*_s_: peak area of the internal standard,*m*_s_: sample volume of the internal standard (μg),*m*: sample weight (g).

#### 2.2.3 GC–IMS analysis

These experiments used the Flavor‐Spec Laboratory Analytical Viewer food flavor analysis and quality control system (GC–IMS system with CTC automatic headspace injector and Library Search qualitative software; FlavourSpec, Gesellschaft für Analytische Sensorsysteme mbH).

Two grams of crushed sample was placed in a headspace vial. Additional parameters were as follows: headspace temperature, 50°C; headspace time, 5 min; rotation speed, 500 rpm; injection volume, 1 mL; vibration speed, 500 r/min; injection needle temperature, 65°C; filling rate, 200 μL/s; and injection rate, 150 μL/s.

The detection conditions included: FS‐SE‐54 quartz capillary column (15 m × 0.53 mm, 1 μm; FlavourSpec, Gesellschaft für Analytische Sensorsysteme mbH); column temperature, 60°C; carrier gas: high purity N_2_ (purity ≥99.999%); IMS temperature, 45°C; carrier gas flow rate program, initial 2.0 mL/min, hold for 2 min, linear increase to 100 mL/min over 20 min, and linear increase to 150 mL/min over 30 min, for a total run time of 30 min.

Used the standard curve of the corresponding volatile compounds in the GC**–**IMS library G.A.S as a reference, and determined the type and content of volatile compounds in the sample.

#### 2.2.4 Sensory analysis

The method of Lykomitros et al. (2016a) was used with slight modifications. Two hundred grams of each sample were ground into a paste, and sensory evaluations were performed by eight assessors. After sniffing each sample, assessors graded the flavor intensity of the roasted aroma, raw bean aroma, dark roast aroma, raw beany aroma, and fatty aroma on a scale from 1 (very weak) to 9 (very strong) and recorded the results. Samples were assessed in triplicate each time and scored for each aroma attribute; the results were then averaged and presented as radar plots.

#### 2.2.5 Data analysis

The GC–IMS data were analyzed using GC × IMS Library Search software and the Reporter, Gallery Plot, and dynamic principal component analysis (PCA) plug‐ins in Laboratory Analytical Viewer software (Gesellschaft für Analytische Sensorsysteme mbH). Origin 2018 (OriginLab) was used for data processing and plotting. Correlation analysis between volatile flavor substances and sensory evaluation data was performed by partial least squares regression (PLSR) using The Unscrambler X 10.4 (CAMO ASA).

## 3 RESULTS AND DISCUSSION

### 3.1 GC–MS analysis of volatile compounds in different varieties of roasted peanuts

Qualitative and quantitative GC–MS results for volatile compounds from different varieties of roasted peanuts are shown in Table 2. A total of 59 volatile compounds were detected and classified into nine major groups: 16 aldehydes (relative content 22.10%), 10 pyrazines (29.46%), 9 ketones (14.51%), 7 esters (1.53%), 2 alkenes (15.43%), 2 acids (0.73%), 7 alcohols (8.98%), 3 alkanes (2.78%), and 3 other compounds (4.49%). The types and contents of volatile compounds differed markedly among different varieties of roasted peanuts. For example, dimethyl disulfide was detected only in LH965, BS, LH618, and KN308, and 2,5‐diethylpyrazine was not found in PH66, FH23, or HY962. These differences potentially explain variations in flavor characteristics among different roasted peanut varieties.

**TABLE 2:** Determination of volatile compounds in roasted peanuts by GC–MS.

Compound name	Cod	Content (μg/100 g)														
Peanut varieties		LH965	BS	LH618	SH5	JTH1	YH19	J572	XH5	WH29	WH23	JH19	FH29	JNH12	SH1	JH1336
Nonanal	A1	3.080	3.284	4.109	3.980	2.403	4.384	3.267	4.387	1.951	4.516	4.462	1.503	2.299	4.761	2.257
Heptaldehyde	A2	0.519	1.673	2.543	0.434	0.761	0.609	0.426	1.475	0.474	0.668	0.994	0.531	0.586	0.760	0.458
Furfural	A3	5.340	5.667	3.270	1.861	6.139	1.565	1.904	2.084	2.477	–	1.283	0.775	1.193	1.705	1.983
3‐Methylbutyraldehyde	A4	3.405	7.052	3.037	–	3.371	–	–	1.681	–	–	–	–	0.427	–	0.274
2‐Heptenal	A5	0.517	8.221	1.102	0.363	6.200	0.435	0.224	0.828	0.778	0.206	0.249	4.532	1.401	0.316	1.348
Hexanal	A6	2.564	29.265	12.543	1.616	8.800	1.913	1.337	6.363	2.649	0.930	2.150	4.817	3.475	2.963	2.561
trans‐2‐Hexenal	A7	–	0.502	0.602	–	0.340	–	–	–	–	–	–	0.329	–	–	–
2‐Methylbutyraldehyde	A8	5.153	10.63	4.988	0.915	5.395	1.131	0.882	1.232	2.101	–	1.767	0.620	0.386	2.025	0.373
Octanal	A9	–	–	–	16.917	23.643	20.842	–	5.543	–	–	0.260	–	–	4.194	–
Benzaldehyde	A10	12.565	26.763	5.983	3.484	8.750	5.439	3.500	5.504	4.819	2.440	2.744	3.876	2.058	4.181	2.678
Phenylacetaldehyde	A11	1.545	7.732	5.869	0.438	4.609	2.492	1.614	0.661	2.029	2.035	1.042	1.079	0.395	2.365	0.474
Decanal	A12	0.900	1.728	0.809	0.506	0.505	0.795	0.297	0.356	0.336	0.449	0.553	0.187	0.247	0.436	0.281
Isobutyraldehyde	A13	–	–	–	–	–	1.567	–	–	–	–	–	0.379	1.145	–	–
Pentanal	A14	1.724	3.625	2.948	–	2.461	0.909	0.705	2.530	1.505	–	1.774	1.294	1.100	1.317	0.938
(Z)‐Dec‐2‐Enal	A15	1.224	2.333	0.845	–	–	–	0.093	0.103	0.077	–	0.076	0.071	–	–	–
2,4‐Decadienal	A 16	1.070	1.150	0.659	0.129	0.612	0.163	0.084	0.085	0.121	0.106	0.055	0.188	0.109	0.087	0.138
2,5‐Diethylpyrazine	B1	3.334	1.879	6.779	4.007	20.303	7.812	17.635	5.321	21.336	4.365	7.963	19.569	2.137	1.246	5.331
Ethylpyrazine	B2	4.905	6.375	2.177	2.100	6.320	2.916	1.243	3.260	2.209	2.644	1.999	0.959	1.169	1.780	1.406
2,5‐Dimethyl pyrazine	B3	17.046	25.24	6.633	18.188	54.622	21.397	8.506	25.405	15.218	13.986	12.341	5.241	8.966	13.289	9.258
2,3‐Dimethylpyrazine	B4	1.922	3.343	1.157	0.799	2.494	1.485	0.271	1.084	0.632	–	0.575	–	0.323	0.505	0.328
2‐Ethyl‐6‐Methyl‐Pyrazine	B5	13.224	33.652	13.583	0.842	5.104	1.660	3.932	14.827	7.113	4.648	6.651	–	3.832	8.385	0.448
3‐Ethyl‐2,5‐dimethylpyrazine	B6	6.381	14.412	3.858	5.055	15.684	8.251	2.148	5.131	3.282	3.265	2.688	0.849	1.657	3.340	1.778
2‐Ethyl‐3,5‐dimethylpyrazine	B7	–	1.860	–	0.563	1.838	1.244	–	0.554	0.457	1.744	0.433	0.196	0.213	0.411	0.297
2‐Isopropyl‐3‐methoxypyrazine	B8	0.983	3.985	1.183	0.257	0.886	0.353	0.211	0.439	0.301	0.373	0.266	0.475	0.363	0.476	0.434
2‐Ethyl‐5‐methylpyrazine	B9	1.603	2.701	4.132	0.438	2.024	0.958	0.253	0.756	0.401	0.502	0.499	0.201	0.338	0.539	0.324
3,4‐Dihydro‐1‐Methyl‐Pyrrolo[1,2‐a]Pyrazine	B10	–	–	–	0.385	1.284	0.859	0.199	0.391	–	0.473	0.264	–	0.257	0.617	0.211
2‐Methyl‐3‐Heptanone	E1	2.147	–	–	–	0.005	–	–	1.147	–	–	3.221	–	0.687	–	–
2‐Methyltetrahydrofuran‐3‐one	E2	0.617	1.370	0.516	–	2.808	0.601	0.252	0.969	0.348	–	0.463	–	0.302	0.653	0.164
3‐Hydroxy‐2‐butanone	E3	0.501	0.815	0.609	0.355	1.451	0.512	0.304	0.534	0.372	0.336	0.588	0.209	0.163	0.706	0.239
3‐Octanone, 2‐methyl‐one	E3	1.633	5.104	6.018	0.195	1.733	0.357	–	1.104	0.611	–	–	1.160	0.689	0.437	0.745
2‐Nonanone	E5	–	–	0.852	0.357	0.372	0.377	0.252	0.565	0.257	0.622	0.322	–	0.225	0.393	0.251
3‐Hydroxy‐2‐methyl‐4‐pyrone	E6	1.575	2.431	1.042	0.219	0.973	0.628	0.261	0.611	0.196	0.685	0.621	–	–	–	–
3‐Hydroxy‐2‐butanone	E7	0.501	0.815	0.609	0.355	1.451	0.512	0.304	0.534	0.372	0.336	0.588	0.209	0.163	0.706	0.239
2‐Decanone	E8	0.963	1.593	0.419	0.136	–	–	0.099	0.202	0.239	0.269	0.139	–	0.087	–	0.076
2′‐Hydroxy‐5′‐methylacetophenone	E9	0.982	–	0.354	0.239	2.078	0.657	0.217	0.289	0.241	0.441	0.317	–	0.093	0.294	0.139
Methyl pyruvate	F1	0.891	1.273	1.471	0.933	2.066	1.077	–	0.687	–	–	0.489	0.541	0.528	0.936	1.004
Benzyl carbazate	F2	–	–	–	–	0.315	–	–	0.438	–	0.173	0.591	0.808	–	–	–
Methyl 1‐Methylpyrrole‐2‐Carboxylate	F3	0.565	1.568	0.484	–	–	–	0.137	0.268	0.100	0.297	0.195	–	0.156	0.318	0.215
Linalyl acetate	F4	5.413	2.232	2.168	0.535	0.715	1.566	0.055	0.088	0.103	0.700	0.071	0.064	–	–	–
Diisobutyl phthalate	F5	–	–	–	0.074	0.056	0.115	–	–	–	0.270	–	–	0.042	0.042	0.164
Methyl hexadecanoate	F5	0.900	–	–	–	–	–	–	–	–	0.127	–	–	–	–	–
Dibutyl phthalate	F7	2.439	–	–	–	–	–	–	0.032	0.140	0.540	–	–	0.135	0.073	0.658
Dipentene	G1	3.508	15.978	26.439	1.522	3.851	1.680	1.005	2.917	0.557	2.977	1.711	1.492	3.235	1.406	3.211
Styrene	G2	0.732	1.958	27.859	3.485	5.122	23.319	6.409	37.695	5.595	6.873	3.342	12.108	6.533	8.924	3.886
Nonanoic acid	H1	2.426	7.532	13.885	0.233	0.257	0.299	–	–	–	–	–	–	0.091	0.129	0.093
Palmitic acid	H1	0.615	0.542	0.270	–	–	–	–	–	–	–	–	–	–	–	–
1‐Pentanol	J1	0.157	3.588	2.103	–	0.809	–	–	0.984	0.656		–	–	0.575	0.782	0.392
Furfuryl alcohol	J2	1.868	4.758	1.716	0.982	5.138	1.426	0.849	2.874	0.794	0.512	1.208	0.367	0.655	2.037	0.684
2,3‐Butanediol	J3	0.259	0.255	0.569	–	0.213	0.392	0.314	1.605	0.252	–	–	–	0.297	0.812	–
Benzyl alcohol	J4	–	0.424	1.164	14.92	11.119	15.55	9.730	12.254	10.540	7.394	5.923	9.355	5.493	7.112	10.666
1‐Pentanol	J5	0.184	0.464	1.055	–	–	–	0.568	1.596	0.833	–	–	–	–	–	–
3‐Methylbutanolide	J6	–	–	2.409	–	4.593	1.563	1.617	3.036	1.616	0.899	1.168	1.328	1.262	3.301	0.925
3‐Decyn‐2‐ol	J7	1.223	3.120	2.530	0.547	1.029	0.526	0.480	1.163	0.476	0.700	0.317	0.347	0.455	1.033	0.506
4‐Methyl‐octane	K1	2.683	8.441	11.551	0.680	1.333	0.608	0.437	1.246	0.844	–	0.974	0.550	0.428	1.046	0.677
2,5‐Dimethylnonane	K2	2.269	6.412	4.701	0.601	2.396	0.877	0.335	0.578	0.361	0.633	0.693	0.420	0.427	0.866	0.700
2,6,7‐Trimethyl‐decane	K3	2.921	9.355	7.508	0.658	1.558	0.822	0.524	1.754	0.930	0.507	0.838	0.607	0.508	1.104	0.908
2‐Ethylfuran	L1	2.951	7.731	0.450	0.414	2.711	0.925	0.283	0.306	0.282	1.350	0.546	–	0.188	0.398	0.185
N‐Methyl pyrrole	L2	2.762	4.252	6.535	4.687	3.085	4.875	5.084	11.47	3.150	5.578	8.754	1.908	3.713	9.022	3.896
Dimethyl disulfide	L3	0.505	1.339	0.523	–	–	–	–	–	–	–	–	–	–	–	–

Compound name	Cod	Content (μg/100 g)														
Peanut varieties		PH66	YH37	FH30	YH18	JNH6	YH65	FH23	JH18	HY962	FH27	KN308	FH17	XH17	FH22	LH917
Nonanal	A1	2.633	3.035	1.673	7.067	2.691	4.112	0.961	4.603	1.903	6.732	0.819	0.827	5.507	2.446	5.427
Heptaldehyde	A2	1.100	0.855	0.518	1.687	0.643	1.088	0.446	0.867	0.958	1.184	0.821	1.171	0.808	1.082	2.321
Furfural	A3	0.793	–	2.663	5.512	1.871	2.813	–	2.417	–	–	–	–	–	1.190	–
3‐Methylbutyraldehyde	A4	–	1.139	–	7.140	–	3.247	–	2.114	0.704	–	4.633	1.366	–	–	–
2‐Heptenal	A5	0.514	0.395	1.817	0.543	1.915	–	3.867	0.416	0.533	0.335	5.381	7.496	1.174	4.058	1.256
Hexanal	A6	2.996	2.858	4.524	5.121	4.042	4.266	5.392	2.885	1.426	1.238	8.501	11.791	2.421	7.744	6.451
trans‐2‐Hexenal	A7	–	–	–	1.124	0.240	0.305	0.183		–	–	–	0.573	–	0.484	–
2‐Methylbutyraldehyde	A8	0.649	1.080	0.546	9.640	1.835	4.176	0.607	2.920	0.716	–	4.541	1.352	–	2.251	2.910
Octanal	A9	2.076	2.675	6.429	9.467	–	–	–	9.330	3.810	3.794	–	–	3.349	–	1.765
Benzaldehyde	A10	2.709	2.380	2.890	4.224	4.024	4.684	2.990	3.645	3.160	3.196	26.512	6.841	5.133	5.885	7.21
Phenylacetaldehyde	A11	–	–	–	3.232	1.298	1.648	0.789	0.980	0.306	0.359	5.529	1.370	1.193	1.576	0.955
Decanal	A12	0.171	0.276	0.237	0.876	0.337	0.602	0.159	0.506	0.232	0.951	–	–	0.475	0.343	0.503
Isobutyraldehyde	A13	–	–	–	–	0.781	–	1.784	0.312	–	–	–	–	1.447	1.278	–
Pentanal	A14	1.388	1.281	0.171	3.384	1.698	1.113	1.283	1.977	0.758	–	1.366	1.896	–	–	–
(Z)‐Dec‐2‐Enal	A15	–	–	–	0.325	0.119	0.216	0.061	0.146	0.076	0.107	–	–	–	–	–
2,4‐Decadienal	1A6	–	0.087	0.181	0.311	0.218	–	0.156	0.124	–	0.101	0.225	0.288	0.076	0.167	0.180
2,5‐Diethylpyrazine	B1	7.227	3.331	1.379	23.017	9.939	15.588	7.556	13.457	10.637	0.294	0.61	0.385	0.422	0.435	1.327
Ethylpyrazine	B2	0.873	0.915	2.035	9.346	2.846	7.267	1.029	3.523	1.289	1.067	3.603	2.202	1.283	1.759	2.198
2,5‐Dimethyl pyrazine	B3	4.273	4.135	13.923	82.898	24.031	33.742	3.665	26.833	2.925	6.761	25.024	9.535	8.501	10.645	11.413
2,3‐Dimethylpyrazine	B4	–	–	0.573	5.081	1.102	2.411	0.812	1.713	0.600	0.263	1.022	0.652	–	0.411	–
2‐Ethyl‐6‐Methyl‐Pyrazine	B5	0.218	0.138	0.838	4.896	1.700	1.545	–	1.837	1.566	2.612	10.391	7.340	3.291	7.308	6.956
3‐Ethyl‐2,5‐dimethylpyrazine	B6	0.571	1.281	2.577	19.612	7.077	8.061	0.963	9.042	0.696	1.361	5.679	1.642	2.238	2.838	2.293
2‐Ethyl‐3,5‐dimethylpyrazine	B7	–	–	0.402	3.953	0.942	1.274	–	0.927	–	0.548	1.060	0.615	0.531	0.887	1.245
2‐Isopropyl‐3‐methoxypyrazine	B8	0.222	0.234	0.351	0.364	0.519	–	0.448	0.399	0.340	0.420	–	0.746	0.340	0.841	0.965
2‐Ethyl‐5‐methylpyrazine	B9	–	0.206	0.422	1.752	0.921	1.169	–	0.694	–	0.297	0.621	0.472	0.437	0.628	0.697
3,4‐Dihydro‐1‐Methyl‐Pyrrolo[1,2‐a]Pyrazine	B10	–	–	0.299	–	–	–	–	0.954	–	0.393	0.411	0.291	0.292	0.527	0.275
2‐Methyl‐3‐Heptanone	E1	–	–	–	0.147	–	–	0.378	–	–	1.021	2.001	0.754	–	–	0.014
2‐Methyltetrahydrofuran‐3‐one	E2	–	0.132	0.311	2.091	0.651	0.715	–	0.658	–	–	–	–	–	0.391	–
3‐Hydroxy‐2‐butanone	E3	0.241	0.408	0.254	1.382	0.538	0.919	0.197	0.654	0.223	–	1.187	0.453	–	–	–
3‐Octanone, 2‐Methyl‐One	E3	0.380	–	0.823	–	1.030	–	0.992	0.440	0.312	0.362	1.715	1.752	0.593	1.926	1.543
2‐Nonanone	E5	0.226	0.199	0.264	1.239	0.436	0.583	–	0.646	–	0.259	–	–	0.232	0.264	0.732
3‐Hydroxy‐2‐methyl‐4‐pyrone	E6	–	0.328	0.329	2.329	0.747	1.915	–	0.815	0.231	0.328	0.432	0.205	0.158	0.497	0.421
3‐Hydroxy‐2‐butanone	E7	0.241	0.408	0.254	1.382	0.538	0.919	0.197	0.654	0.223	–	1.187	0.453	–	–	–
2‐Decanone	E8	0.078	0.099	–	1.703	0.937	0.740	–	–	–	–	–	–	0.135	–	0.266
2′‐Hydroxy‐5′‐methylacetophenone	E9	–	–	0.170	1.040	0.536	1.788	–	0.492	0.114	0.226	0.373	–	0.128	0.268	1.952
Methyl pyruvate	F1	0.415	0.949	1.233	1.111	1.513	0.893	0.796	0.836	0.501	0.367	1.521	0.612	1.004	0.733	–
Benzyl carbazate	F2	–	–	–	0.518	0.691	0.568	–	0.343	0.229	–	0.463	0.246	–	–	0.901
Methyl 1‐Methylpyrrole‐2‐Carboxylate	F3	–	–	0.321	0.425	0.572	–	–	0.405	–	0.179	0.254	0.117		–	–
Linalyl acetate	F4	–	–	–	–	–	–	0.161	0.785	0.377	–	–	–	–	0.464	0.757
Diisobutyl phthalate	F5	0.037	0.108	0.072	0.522	0.068	0.184	0.037	0.308	0.071	0.147	–	0.259	–	0.062	–
Methyl hexadecanoate	F5	–	–	–	0.251	–	0.604	–	0.077	–	0.043	–	–	–	–	–
Dibutyl phthalate	F7	–	–	–	0.963	–	–	0.177	0.095	0.117	0.352	–	0.106	0.257	0.248	0.202
Dipentene	G1	0.577	1.997	2.646	1.722	2.895	4.821	1.734	1.592	1.321	1.909	0.583	1.213	0.773	1.704	4.145
Styrene	G2	22.181	5.858	6.035	27.387	13.535	15.707	5.071	37.314	25.720	22.028	29.122	23.801	24.988	62.168	75.872
Nonanoic acid	H1	–	–	–	0.314	0.173	0.265	–	–	–	0.133	–	–	–	–	–
Palmitic acid	H1	–	–	–	0.304	0.118	1.661	–	0.267	0.571	0.044	–	0.303	–	–	0.157
1‐Pentanol	J1	0.881	0.423	0.933	0.893	0.733	0.618	–	0.383	0.211	–	0.685	0.611	0.280	0.942	2.283
Furfuryl alcohol	J2	0.455	0.651	0.803	4.009	1.289	2.065	–	2.028	–	0.285	0.912	0.509	0.506	1.377	1.047
2,3‐Butanediol	J3	0.459	0.562	–	–	–	–	–	0.547	0.151	–	–	–	–	0.853	1.066
Benzyl alcohol	J4	6.375	3.394	9.559	13.209	6.712	2.233	9.457	3.565	3.527	8.399	5.688	2.584	9.086	7.186	13.436
1‐Pentanol	J5	0.005	–	–	–	–	0.012	–	0.021	–	–	0.004	–	0.116	–	–
3‐Methylbutanolide	J6	0.999	0.479	1.705	3.229	1.423	1.759	0.995	0.815	–	–	–	–	–	1.896	1.714
3‐Decyn‐2‐ol	J7	0.834	0.772	0.332	1.061	0.914	–	0.399	0.761	0.524	–	–	0.319	0.617	1.334	1.987
4‐Methyl‐octane	K1	0.953	1.269	0.513	1.523	0.539	1.446	0.414	–	0.542	–	0.794	1.060	–	–	–
2,5‐Dimethylnonane	K2	0.561	0.807	0.642	1.092	0.723	1.473	0.282	1.078	0.333	0.325	–	0.581	0.269	0.841	1.273
2,6,7‐Trimethyl‐decane	K3	0.865	1.267	0.618	1.351	0.834	1.431	0.469	1.130	0.494	0.386	0.734	0.762	0.301	1.191	1.825
2‐Ethylfuran	L1	–	0.103	0.399	1.591	1.177	1.535	–	0.696	–	0.378	2.869	–	0.254	0.748	0.247
N‐Methyl pyrrole	L2	4.724	4.142	3.649	10.744	4.762	6.087	1.673	10.001	3.317	3.698	2.421	2.341	4.049	4.282	13.104
Dimethyl disulfide	L3	–	–	–	–	–	–	–	–	–	–	0.819	–	–	–	–

Previous studies have found that pyrazines have the highest relative abundance among the volatile compounds of roasted peanuts. They are formed by condensation of α‐amino ketones from the products of the Strecker degradation reaction (i.e., the reaction of amino acids with α‐dicarbonyl compounds, which lose a molecule of CO_2_ and degrade into aldehydes and amino ketones with one less carbon atom). The various special aldehydes produced by this reaction are called Strecker aldehydes and are one of the factors responsible for different food aromas (Cho et al., 2010; Scalone et al., 2015). In this study, we detected a total of 10 pyrazines, and the average content of pyrazines was approximately 41.483 μg/100 g. The most common pyrazines were 2,5‐diethylpyrazine, ethylpyrazine, 2,5‐dimethyl pyrazine, and 3‐ethyl‐2,5‐dimethylpyrazine, and 2,5‐diethylpyrazine had the highest average content of 7.487 μg/100 g (0.294–23.017 μg/100 g). Studies have reported that substituted pyrazines such as ethylpyrazine, 2,5‐dimethyl pyrazine, and 3‐ethyl‐2,5‐dimethylpyrazine have sufficiently low thresholds to make them characteristic flavor substances of roasted peanuts (Ho et al., 1982).

Aldehydes are another abundant volatile compound in roasted peanuts (Scalone et al., 2015). Because of their high content, variety, and low threshold for categorization as a flavor substance in roasted peanuts, these compounds tend to have a greater impact on overall flavor than pyrazines (Dun et al., 2019). Sixteen aldehydes were detected in the 30 roasted peanut varieties, and the average content of aldehydes was 31.125 μg/100 g. Nonanal, heptaldehyde hexanal, and benzaldehyde were common in all tested samples, and benzaldehyde had the highest mean content of 6.009 μg/100 g (2.058–26.763 μg/100 g). The other 12 aldehydes did not co‐occur in all samples, and their contents varied greatly among peanut varieties. There are several possible explanations for this variation. Aldehyde content has been linked not only to roasting temperature (Zhang et al., 2018) but also to differences in fatty acid composition of peanuts (Jia et al., 2021; Sithole et al., 2022). Kaneko et al. (2013) identified five common aldehydes among the volatile components of roasted in‐shell peanuts (propanal, benzaldehyde, nonanal, furfural, and 2‐heptenal) and found strong correlations between aldehyde types and contents and the storage time of raw peanut material. One of these factors could therefore explain the variance observed in the samples tested here. In addition, Chetschik et al. (2010) reported that phenylacetaldehyde was the main off‐flavor substance in roasted peanuts. Here, although phenylacetaldehyde was not the most prominent aldehyde detected, it was present in 27 of 30 varieties, suggesting that it does play a role in the flavor composition of the tested varieties.

One furan (2‐ethylfuran) and one ketone derivative of furan (2‐methyltetrahydrofuran‐3‐one) were also detected. Furans and their derivatives are present in the volatile flavor substances of many hot processed nuts and oils, where they originate from the high‐temperature cracking of sugars (Dun et al., 2019). These two substances were not present in all varieties of roasted peanuts (2‐ethylfuran was not found in FH29, PH66, FH23, HY962, or FH17), and the reasons for this may be related to amino acid composition, roasting temperature, or roasting time (Cantalejo, 1997).

A variety of alkenes, acids, esters, alcohols, ketones, and alkanes were also detected in the 30 roasted peanut varieties, with the most common substances being dipentene, styrene, 2,6,7‐trimethyl‐decane, and *N*‐methyl pyrrole. Alkenes, esters, and alcohols (e.g., dipentene, styrene, linalyl acetate, and benzyl alcohol) are natural flavor substances of peanuts, and differences in the types and contents of these compounds distinguish peanut varieties (Jiao et al., 2016). Acids are mainly derived from hydrolysis of peanut fatty acids, and differences in their contents and types across tested peanut varieties were related to storage time and storage methods of raw peanut materials (Raigar et al., 2017). Ketones and alkanes are mainly derived from the Maillard and caramelization reactions (Boateng & Yang, 2021). Roasted peanuts are rich in alkenes, acids, esters, alcohols, and alkanes (as this study shows), but their thresholds are high and their contribution to the overall flavor of roasted peanuts is generally considered to be minimal (Wei et al., 2012). The numerous volatile compounds of peanuts have specific characteristic aromas, and the aroma of roasted peanuts is not dominated by one or a few compounds but instead reflects the synergistic effect of multiple components (Lykomitros et al., 2016b). The GC–MS detection of multiple volatile compounds with different degrees of prominence in 30 roasted peanut varieties reinforces previous findings while also demonstrating the need to further characterize and quantify these volatiles to assess their effects on the overall flavor profile.

### 3.2 GC–IMS analysis of volatile compounds in different varieties of roasted peanuts

**FIGURE 1:**
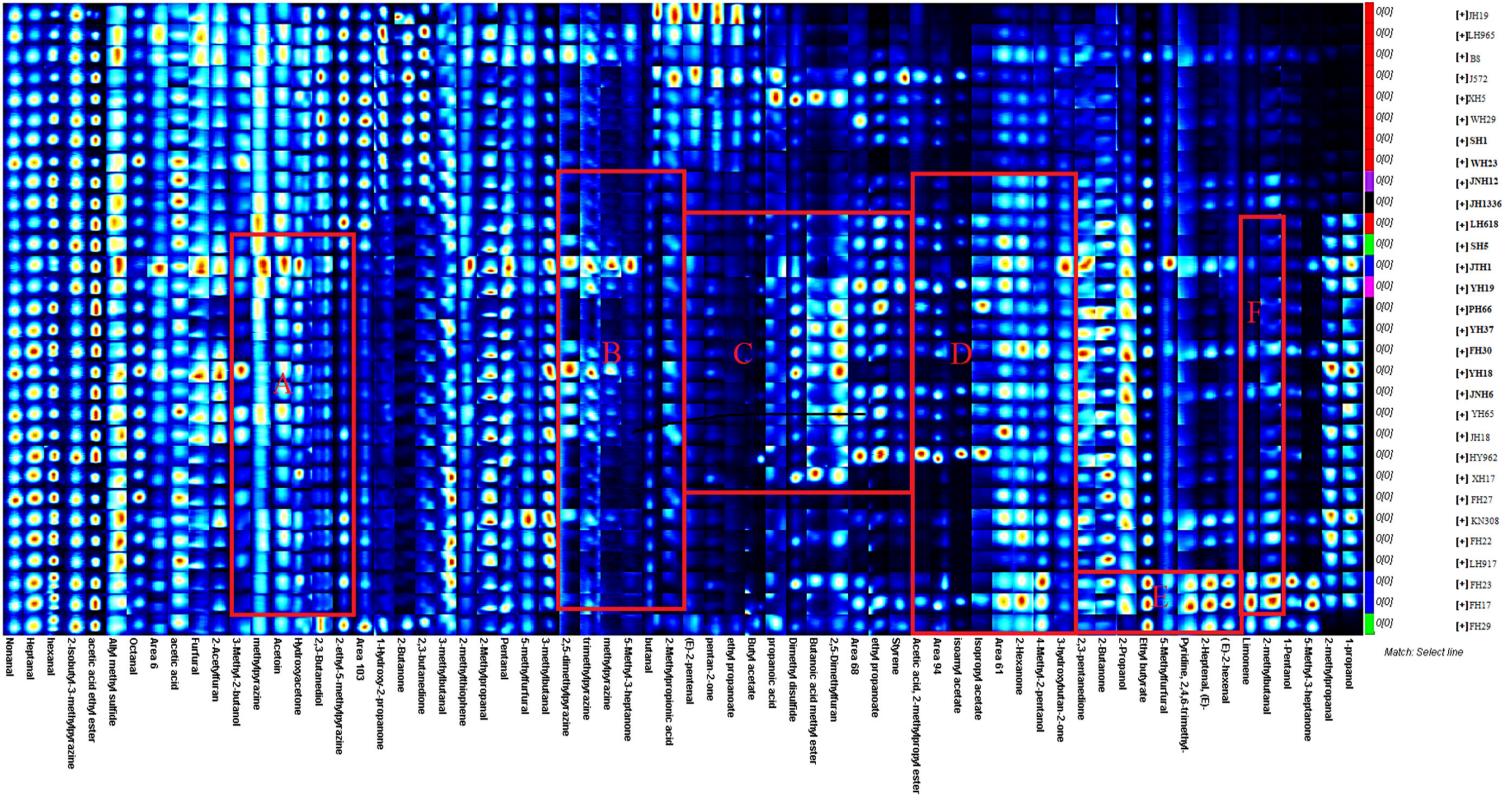
Gallery plot of volatile compounds in roasted peanuts.

**FIGURE 2:**
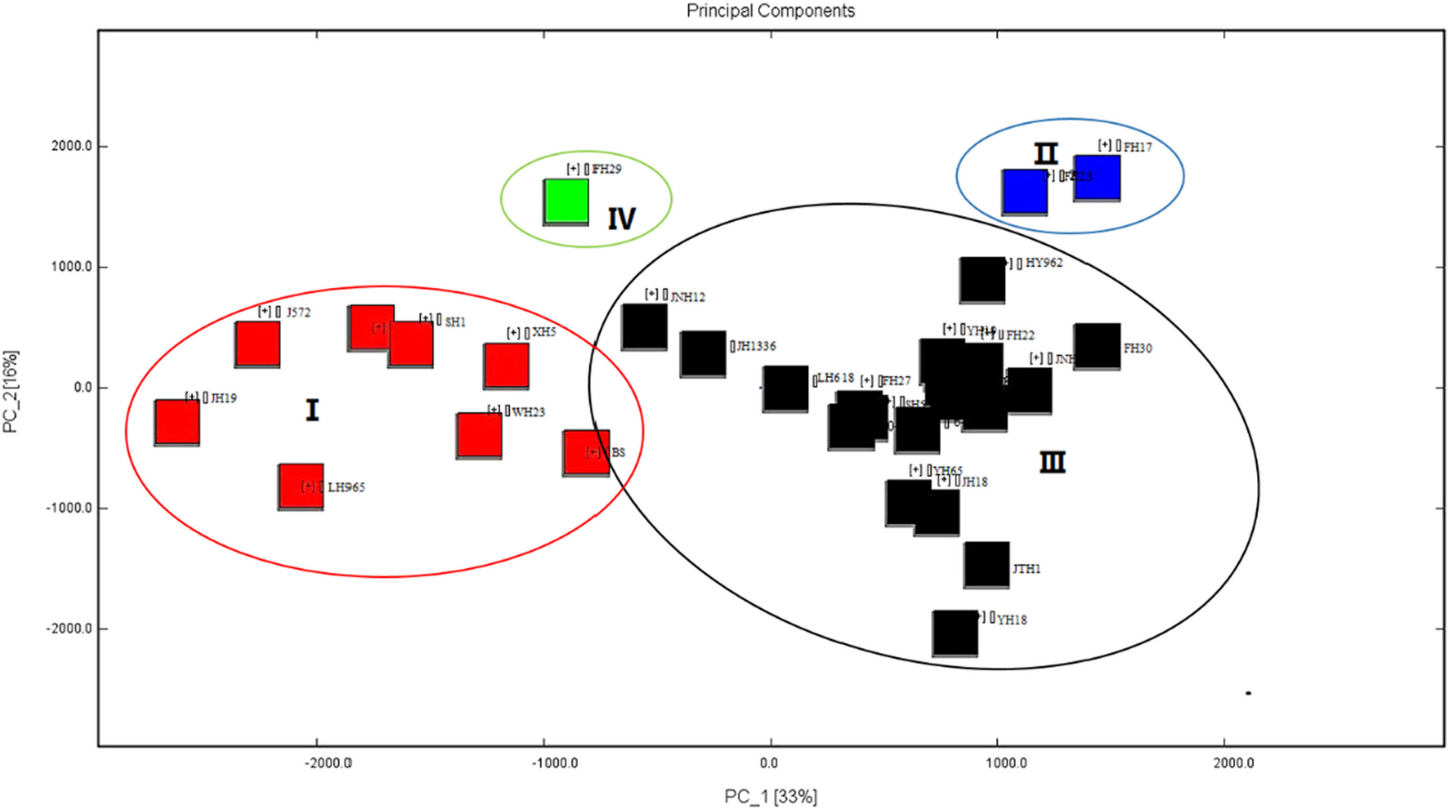
PCA of volatile compounds in roasted peanuts.

GC–IMS was used to further characterize the volatile compounds of the tested peanut varieties. This technique revealed a total of 61 volatile compounds (Figure 1). Similar to the GC–MS analysis results, it can be divided into 9 categories, including 17 aldehydes, 5 pyrazines, 12 ketones, 9 esters, 2 olefins, 3 acids, 6 alcohols, 3 alkanes, and 4 other compounds. The difference lies in the types of volatile compounds, especially in the significant differences in the results of the two detection methods for pyrazine compounds. GC‐IMS detected fewer types of pyrazine compounds than GC–MS detection results. Moreover, 2,3,5‐trimethylpyrazine and 2‐methylpyrazine were not detected in GC–MS, which may be due to the different sensitivities of GC‐IMS and GC–MS technologies in detecting substances. The substances detected by GC‐I MS tend to be compounds with smaller molecular weights. PCA was performed on the results, and the combined contribution of the first two principal components was 85%. As shown in Figure 2, the peanut varieties BS, J572, XH5, WH29, WH23, JH19, and SH1 were clustered into Group I, and most of the remaining samples were clustered into Group III.

On the basis of the PCA results, all the peaks identified in the GC–IMS spectra were selected, and the corresponding visual maps were combined side‐by‐side using the Gallery Plot plug‐in to generate characteristic flavor fingerprints of the roasted peanut varieties. As shown in Figure 1, region A included compounds such as 2,3‐butanediol, 2‐ethyl‐5‐methylpyrazine, 1‐hydroxy2‐propanone, and 2‐butanone that were evident in peanuts from Groups I and IV but less so in peanuts from Groups II and IV. Region B included compounds such as 2‐methylpropionic acid, butanal, trans‐2‐pentenal, 2‐pentanone, ethyl propionate, and butyl acetate that were present in Classes I and IV but less abundant in Groups II and III. Region C included propionic acid, methyl butyrate, ethyl propionate, isobutyl acetate, isopropyl acetate, isoamyl acetate, 2,5‐dimethylfuran, styrene, and other compounds; these were more abundant in Group III as a whole, as well as some samples from Groups I, IV, and II. Their abundance was lower in other peanut samples (LH965, BS, WH23, JH19, JNH12, SH1, JH1336, FH27, KN308, FH22, and LH917). Region D included compounds such as 2‐hexanone, 4‐methyl‐2‐pentanol, 3‐hydroxy‐2‐butanone, 2,3‐pentanedione, 2‐butanone, 2‐propanol, and ethyl butyrate that were present in Groups II, III, and IV but less abundant in Group I. Region E included compounds such as 5‐methyl furfural, 2,4,6‐trimethylpyridine, hexanal, trans‐2‐hexenal, dipentene, 2‐methylbutyraldehyde, 1‐pentanol, and 5‐methyl‐3‐heptanone that were present mainly in Groups I and IV peanuts, present at low levels in BS, JTH1, FH30, JNH6, KN308, and FH22, and nearly absent in the rest of the samples. Region E included two compounds, 1‐hydroxy2‐propanone and 1‐propanol, that were present mainly in Group III peanuts, present at very low levels in BS, WH23, JNH12, and JH1336, and not detected in LH965, J572, XH5, WH29, JH19, FH29, and SH1. There was relatively little variability in the types of roasted flavor substances within the same group and greater variability between groups. Differences in flavor content within the same group of peanuts could also be distinguished by the gas‐characteristic flavor fingerprint of roasted peanuts. For example, samples with high contents of methyl butyrate and 2,5‐dimethylfuran were YH19, XH5, PH66, YH37, FH30, YH18, JNH6, YH65, and XH17, and those with high contents of ethyl propionate, styrene, isobutyl acetate, isopropyl acetate, and isoamyl acetate were LH618, JTH1, YH19, J572, HY962, and FH17, by contrast, the latter five chemicals were not detected in varieties LH965, BS, WH23, JH19, YH18, FH27, XH17, and LH917. Thus, flavor substance fingerprints produced by GC–IMS could visually reveal obvious differences in volatile flavor substances among 30 different roasted peanut samples. Li et al. (2019) obtained flavor substance fingerprints and combined the results with PCA to classify different varieties of pine mushrooms. Likewise, Zhao et al. (2021) used GC–MS and GC–IMS to create flavor substance fingerprints for the classification of representative kiwifruit varieties. Chen et al. (2020) used GC–IMS to analyze volatile flavor components of 122 Chinese yellow wine samples from three regions of China, determining the origin of the wine raw materials by fingerprinting volatile flavor components. The results of these studies demonstrate the feasibility of analyzing flavor substances in food products by GC–IMS to determine their varieties.

### 3.3 Correlation analysis between volatile compounds and flavor attributes of roasted peanuts

#### 3.3.1 Sensory analysis of roasted peanuts

**FIGURE 3:**
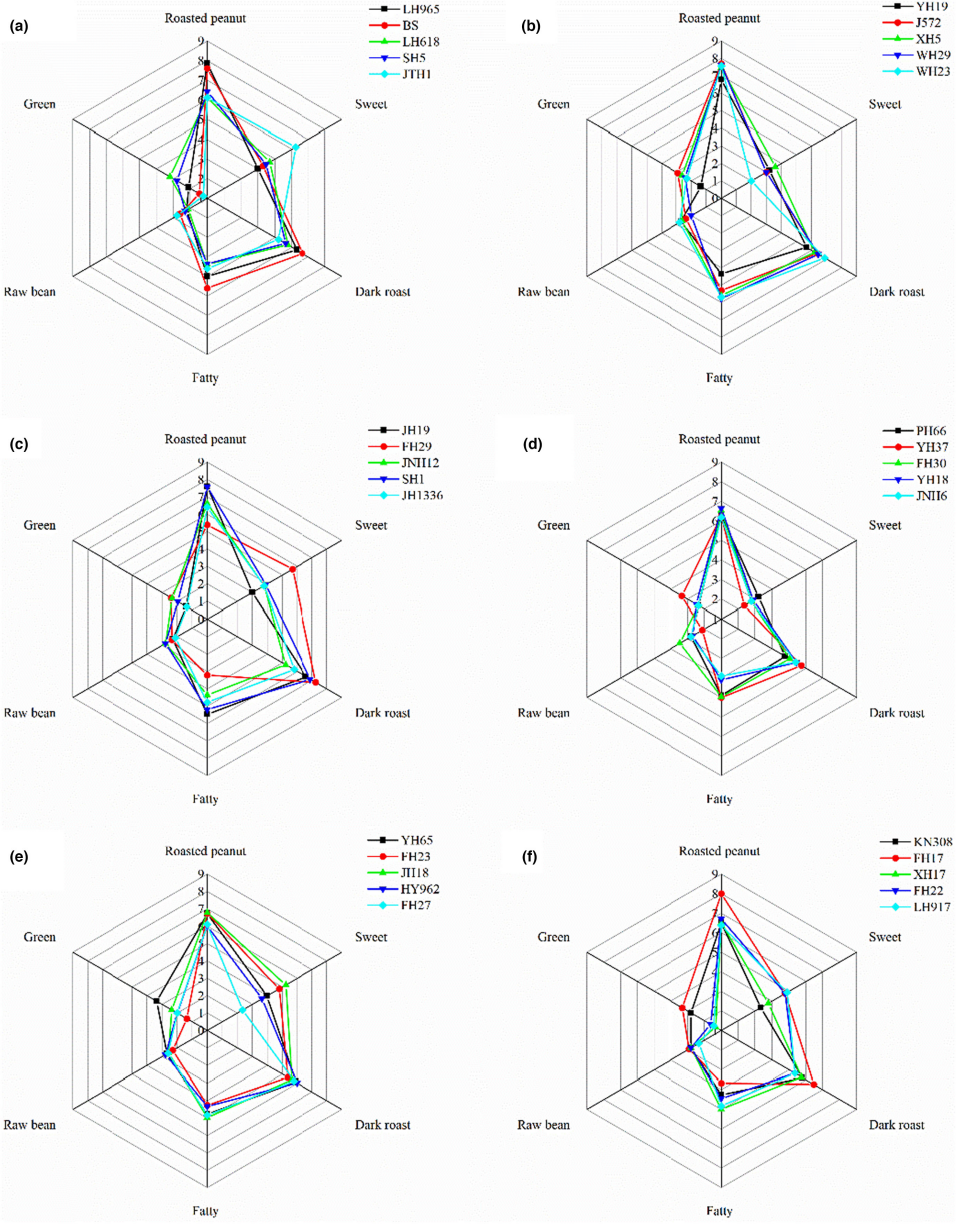
Radar map of sensory evaluation of roasted peanuts.

The main flavor attributes of roasted peanuts are roasted peanut aroma, sweet aroma, dark roast aroma, fatty aroma, raw bean aroma, and green aroma. In this study, each attribute was analyzed by eight sensory assessors to obtain a descriptive sensory evaluation of the 30 roasted peanut varieties (Figure 3). In terms of overall scores, roasted peanut aroma and dark roast aroma were the main flavors of roasted peanuts, with LH965 scoring highest in roasted peanut aroma and FH29 scoring highest in dark roast aroma. By contrast, the flavor intensity of green aroma was relatively low, and the differences in scores between varieties were more pronounced, probably because green aroma is mainly derived from aldehydes (Zhang et al., 2022) and the type and content of aldehydes varied greatly among the 30 varieties.

#### 3.3.2 Partial least squares regression (PLSR) analysis between volatile compounds and flavor attributes

**FIGURE 4:**
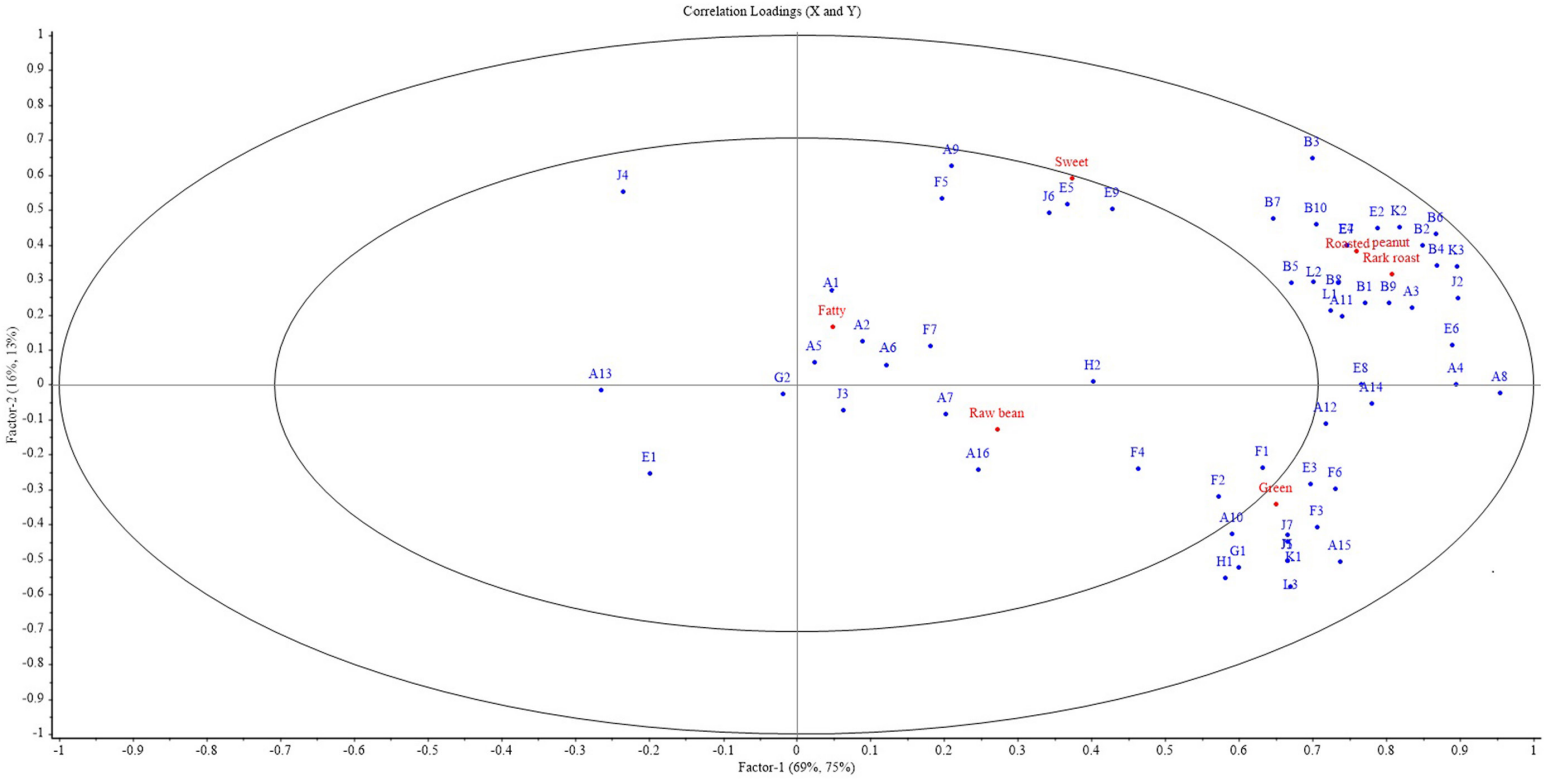
PLSR correlation between volatile compounds and sensory attributes. Blue points indicate volatile compounds as defined in Table 2.

To determine the correlations between volatile compounds and flavor attributes of roasted peanuts, we performed PLSR analysis with volatile compound contents of different varieties determined by GC–MS analysis as the independent variable and sensory analysis scores as the dependent variable (Figure 4). The X matrix was designed to represent volatile compounds, and the Y matrix was designed to represent sensory attributes. Two main components were extracted that explained 85% of the variance in X (volatile compounds) and 88% of the variance in Y (sensory attributes). The sum of the variance explained by the first two principal components was greater than 80%, and most of the points were located between the two ellipses, indicating successful model building.

Pyrazines are one of the most important flavor substances in baked goods; these compounds have good flavor dispersion and a low threshold, producing strong roasted and nutty flavors (Baker et al., 2003). Previous studies have suggested that pyrazines have a positive effect on roasted peanut aroma and dark roast aroma; make an important contribution to the aroma of roasted foods (Spreng et al., 2021); and are essential flavor substances for roasted peanuts (Mason et al., 1966). 2,5‐Diethylpyrazine may be the most relevant volatile compound for the main flavor of roasted peanuts, and ethyl‐substituted pyrazines have a low odor threshold and are highly detectable at low concentrations (de Freitas Floriano et al., 2020). Thus, differences in the types and levels of pyrazines in roasted peanuts may be responsible for differences in the characteristic aromas of peanut varieties after heating.

Aldehydes are the predominant nonheterocyclic volatile constituents in roasted peanuts and are mainly derived from oxidation, degradation of oils and fats, and Strecker degradation reactions. Prior to the discovery of pyrazines, aldehydes were thought to be the main flavor substances in roasted peanuts (Mason et al., 1966). As can be seen from Figure 4, different types of aldehydes were strongly correlated with the six flavor attributes of roasted peanuts. Furfural and phenylacetaldehyde exhibit mainly nutty, grilled, and caramelized odors, and they were positively correlated with roasted peanut aroma and dark roast aroma. Octanal mainly manifests as apricot sweetness, and it was positively correlated with roasted peanut sweet aroma properties. Nonanal, heptaldehyde, and 2‐heptenal have waxy, fatty aromas and were positively correlated with fatty aroma in roasted peanuts. Hexanal and trans‐2‐hexenal possess strong fruity aromas and are positively correlated with roasted peanut raw bean aroma. Hexanal is also considered to be the main aldehyde present in fresh peanuts (Pattee et al., 1969). All the above aldehydes are medium relative molecular mass aldehydes (C6–C9) with low threshold levels and high flavor rendering capacity (Ruosi et al., 2012).

Three volatile compounds, 2‐ethylfuran, 2‐methyltetrahydrofuran‐3‐one, and *N*‐methyl pyrrole, were also positively correlated with roasted peanut aroma and dark roast aroma, the main flavors of roasted peanuts. Furans and their ketone derivatives mainly contribute caramelized and nutty flavors to roasted and fried foods (Liao et al., 2019). Pyrroles provide a burnt odor and are one of the sources of burnt flavor produced by roasting peanuts at high temperatures. These compounds, although not very diverse and not very abundant, contribute significantly to the main flavor of roasted peanuts (Lee et al., 2020). Ketones, esters, and alkenes have mainly fruity and green aromas and contribute significantly to the green aroma of roasted peanuts. The low sensitivity of the human sense of smell to these volatile compounds makes green aroma the flavor attribute with the lowest flavor intensity (Alasalvar et al., 2010). Other acids, alcohols, and alkanes also showed some correlation with the flavor attributes of roasted peanuts: 2,5‐dimethylnonane, 2,6,7‐trimethyl‐decane, and furfuryl alcohol with roasted peanut aroma, dark roast aroma, and green aroma; nonanoic acid with green aroma; and styrene with fatty aroma. This may be because certain compounds are positively correlated with specific organoleptic properties and their presence is related to a chemical reaction in the sample that is responsible for production or enhancement of other aromatically active compounds, even if the original compound itself is odorless.

## 4 CONCLUSION

In the present study, we qualitatively and quantitatively analyzed the characteristic volatile compounds of 30 roasted peanut varieties by GC–MS. The GC–IMS technique was used to obtain gas fingerprints of the characteristic flavor profiles of roasted peanuts and to classify them according to their volatile flavor substances. Correlations between roasted peanut flavor attributes and volatile compounds were analyzed by PLSR to identify the characteristic flavor substances of roasted peanuts. Zines compounds such as 2,5‐dimethylpyrazine and 2,5‐diethylpyrazine are the basic flavor substances of roasted peanuts, endowing roasted peanuts with roasted peanuts and dark roasted peanuts. Aldehydes are the most diverse flavor compounds in roasted peanuts, and have a certain correlation with the six flavor properties of roasted peanuts. Furan and its ketone derivatives, as well as pyrrole, are positively correlated with roasted peanut aroma and dark coast aroma. Ketones, esters, and olefins contribute significantly to the green aroma properties of roasted peanuts. The types and contents of acids, alcohols, and alkanes in roasted peanuts vary greatly, and they also show a certain correlation with the various flavor properties of roasted peanuts. The results of this study will be useful for identifying peanut varieties and selecting specific varieties for processed products.

## AUTHOR CONTRIBUTIONS

**Liangchen Zhang:** Data curation (lead); methodology (lead); writing – original draft (lead). **Puxiang Shi:** Data curation (equal); resources (lead). **Jian Sun:** Data curation (equal); formal analysis (equal); software (lead); writing – original draft (equal). **Mengxi Xie:** Methodology (equal); validation (equal). **Haixin Wang:** Resources (equal); validation (equal). **Taiyuan Shi:** Conceptualization (equal); funding acquisition (lead). **Miao Yu:** Conceptualization (lead); funding acquisition (equal); project administration (equal); writing – review and editing (lead).

## CONFLICT OF INTEREST STATEMENT

The authors declare that they have no known competing financial interests or personal relationships that could have appeared to influence the work reported in this paper.

## Data Availability

The data that support the findings of this study are available on request from the corresponding author.
